# Some Applications of Isotope Analysis of Lead in Food by ICP-MS

**DOI:** 10.6028/jres.093.120

**Published:** 1988-06-01

**Authors:** H. M. Crews, R. C. Massey, D. J. McWeeny, J. R. Dean, L. Ebdon

**Affiliations:** Food Science Laboratory, Ministry of Agriculture, Fisheries and Food, Norwich, U.K.; Department of Environmental Science, The Polytechnic, Plymouth, U.K.

## Introduction

The lead concentration in food items is usually low, but accurate measurements of these concentrations are necessary if useful estimates of dietary intake are to be made. Measurements of lead at around 10 ng/g are usually beset by the two problems of sensitivity and, more importantly, matrix effects when atomic absorption techniques are employed. The advent of instruments in which samples are aspirated into an ICP torch (as an ion-source) interfaced with a mass spectrometer (as an ion-counter) open up new possibilities for the accurate measurement of lead in food at normal levels.

The present work explores the relative merits of two approaches, viz., standard additions and isotope dilution analysis (IDA). The latter relies upon the fact that in ICP-MS, measurements are made on a mass-by-mass basis and therefore each isotope of an element is measured separately. Where an isotope is normally present in food at low abundance, addition of a known amount of the element artificially fortified with this isotope and measurement of the ratio of the abundance of this isotope as compared to one of those which has not been fortified (IDA), will allow calculation of the amount of analyte present in the sample. The techniques have been assessed on samples of wines and aqueous slurries of a food powder (dried milk).

Satisfactory measurement of isotope ratios under these circumstances indicates the potential of IDA by ICP-MS for: a) accurate measurement of lead at low levels in food; b) metabolic and nutritional studies; and c) assessment of geographical origin of food.

## Experimental

All measurements were made with a VG Plasmaquad. Solutions were pumped at 1 mL min^−1^ into a fixed cross-flow nebulizer (Jarrell-Ash) fitted into a water-cooled Scott-type double-pass spray chamber maintained at 10±3 °C.

For isotope dilution analyses, samples were enriched with ^206^Pb at 5 ng·mL^−1^; for standard additions, samples were spiked with 0, 1, 5 or 10 ng·mL^−1^ natural Pb. Milk powders were aspirated as a slurry in Triton X-100 [1].

## Results and Discussion

**Figure 1:**
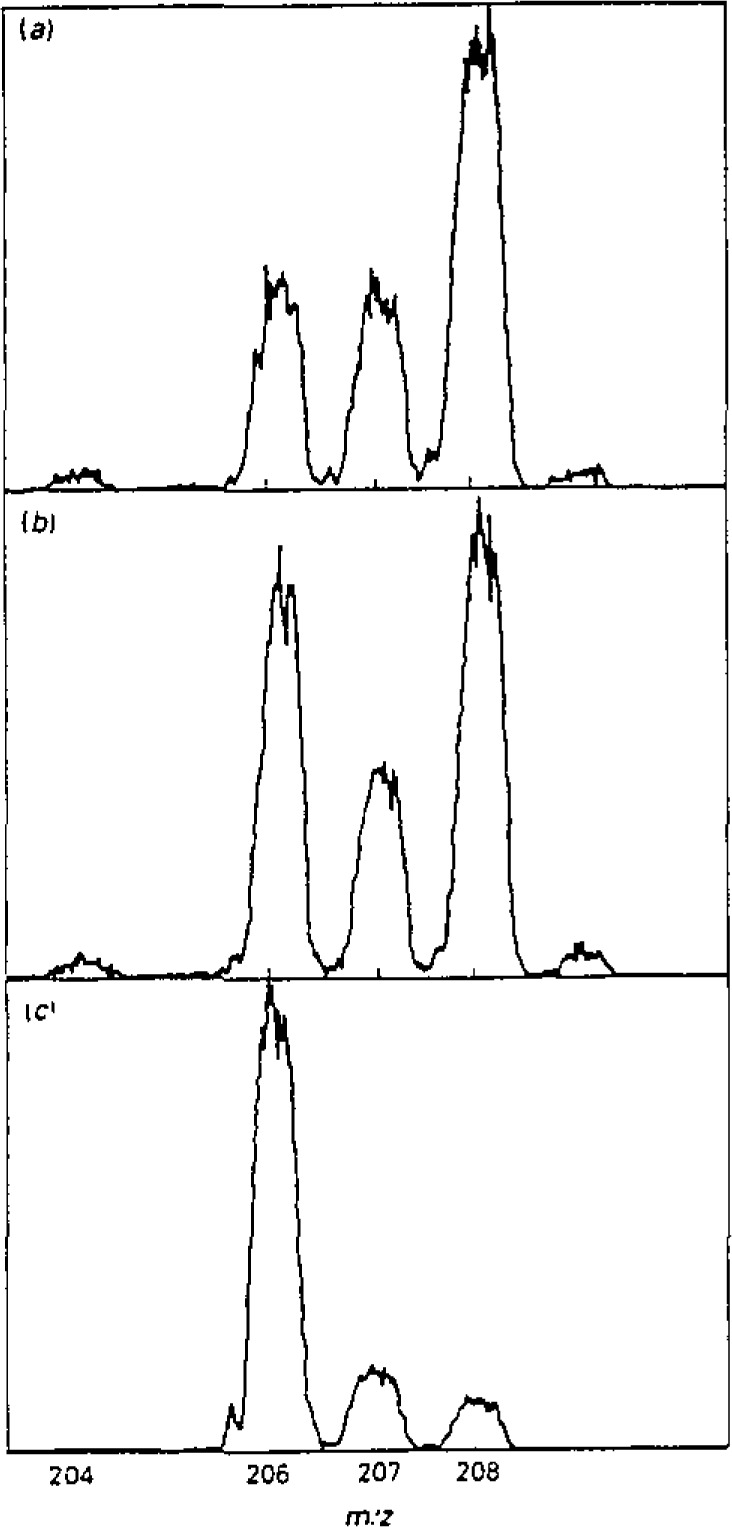
Mass spectra of 10 ng mL^−1^ Pb: (a) Natural Pb; (b) Lead and (c) enriched ^206^Pb.

Comparison of isotope concentration as measured by repeated scanning from 203 to 210 *m*/*z* and by “peak-hopping,” i.e., repeated measurements at each mass unit in turn showed that the former usually gave a better precision. Although the peak-hopping routine allowed more time to be spent in measuring the low-abundance isotopes and hence might be expected to arrive at a more precise assessment, this advantage was offset by the inaccuracy due to noise at the peak crest (fig. 1). Since peak-hopping is essentially based on peak-height measurement it proved somewhat less precise than the peak-area based scanning mode. Typically the coefficients of variation for measurements of 208/206 at 10 ng·mL^−1^ were around 0.5% for scanning mode as compared to 2.5% by peak-hopping (table 1). However the latter was conducted in 13 seconds as compared to 130 seconds; if a slightly impaired precision is acceptable the speed of throughput by peak-hopping may be attractive. Recently improved software may make the precision more nearly comparable to that from scanning.

The accurate and precise measurement of lead isotope ratios (table 2) after correction for mass discrimination using NBS SRM 981 Lead enabled the technique of IDA to be used to measure the lead content of dried milk powders and of wine (table 3). The accuracy of IDA was confirmed by standard additions, small differences in concentration at the ng·g^−1^ level were observed and this is of importance for clinical and nutritional studies.

The 207/206 lead ratio of lead from the Broken Hill area of Australia is reported at 0.960. This value is consistent with the data reported for the Australian milk powder (table 2). There is some indication that the Gawler River Valley wine reflects this ratio but this needs to be confirmed. None of the Italian wines show evidence of contamination by lead originating from Broken Hill. Fuller details of this and related studies are published elsewhere [2].

## Summary

1. *The accuracy and precision* achievable using scanning and peak modes for measurements of lead isotope ratios were studied. If sufficient time and sample materials were available scanning gave better accuracy and precision (table 1).
2. *Isotope ratios* in milk powders and wines of European and Australian origin have been compared. Australian milk powder has a 207/206 lead ratio consistent with that determined for lead in the Broken Hill area of Australia (table 2).
3. *Isotope dilution analysis* (*IDA*) gave more precise data than standard additions as a means of measuring total lead content in milk powder slurries and wine. Accurate and precise data for both matrices were obtained using IDA-ICP-MS (table 3).

## Figures and Tables

**Table 1:** Measurement of 10 ng mL^−1^ Pb (SRM 982), 208/206 ratio using scanning and peak hopping modes

	Scanning^a^	Peak hopping^b^
Certified	1.000	1.000
Measured mean (*n* = 10)	1.082	1.098
RSD, %	0.5	2.6
95% Confidence limits of ratio	±0.003	±0.022

a 480 sweeps; 512 channels 500 μs/channel.

b Dwell time/mass ∝ % abundance; total run 13 s.

**Table 2:** Measurement of lead isotope ratios in milk powder slurries and wines using scanning mode

	208/206	(% RSD)	207/206	(% RSD)
Certified (SRM 981)	2.168		0.915	
*Milk Powders*				
European	2.063	(8.0)	0.890	(7.0)
Australian	2.167	(7.6)	0.959	(11.3)
*Italian Wines*				
Burola 1975	2.121	(2.6)	0.858	(2.0)
Barola 1978	2.243	(1.4)	0.873	(1.3)
Barola 1979	2.154	(2.2)	0.854	(2.1)
Borgogoni 1979	2.137	(0.9)	0.873	(0.7)
*Australian Hines*				
Gawler River Valley 1983	2.164	(2.5)	0.940	(2.7)
Hunter Valley 1985	2.090	(2.7)	0.849	(4.0)

**Table 3:** Lead concentration in milk powder (ng g^−1^) and wine (ng mL^−1^)

Sample	Isotope Dilution Analysis	Standard Additions
*Milk Powder*		
European	21.±09^a^	30±16^a^
Australian	16.2±1.5	33±16
*Italian Wines*		
Barola 1975	92.7±2.7	106±9.5
Barola 1978	83.8±1.2	94±3.5
Barola 1979	101.4±3.7	99±8
Borgogoni 1979	143.4±2.5	150±10
*Australian Wines*		
Gawler River Valley	49.8±1.1	56±4
Hunter Valley	30.8±1.0	42±4.5

a ±1 standard deviation.
